# Walk, talk, think, see and feel: harnessing the power of digital biomarkers in healthcare

**DOI:** 10.1038/s41746-024-01023-w

**Published:** 2024-02-24

**Authors:** Dylan Powell

**Affiliations:** Faculty of Health Sciences & Sport, University of Stirling, Stirling, UK

**Keywords:** Diagnostic markers

## The critical role of biomarkers

**Fig. 1:**
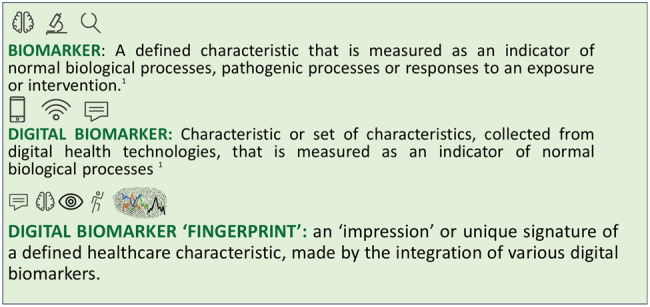
Defining biomarkers with a suggested definition for a digital biomarker ‘fingerprint’. Parts of this figure utilises photos from Stirling University Brand Bank (https://www.stir.ac.uk/brand-bank/visual-assets/) all licensed under CC BY-SA.

A key pillar of healthcare’s journey towards precision medicine has been the proliferation and development of biomarkers to detect, monitor and manage disease. Over the last fifty years, healthcare has seen a rapid acceleration in the discovery of traditional biomarkers for a variety of conditions, including cancer, namely Carcinoembryonic antigen (CEA), and in the 1980s, CA15-3 for breast cancer^1^ (Fig. 1).

Biomarkers remain a crucial source of data and insight for early detection and diagnosis, assessing the response of treatment and prognosis^1,2^. However, with both the complexity and increasing demands for healthcare, research is increasingly exporting novel opportunities that augment and build upon traditional biomarker approaches^2,3^.

## Progressing to digital biomarkers

‘Digital Biomarkers’ (DBx) have emerged as a promising paradigm in healthcare to aid the diagnosis, monitoring, and treatment of various health conditions.

The opportunity has been enabled partly due to a rapid increase in the volume, velocity and variety of data collected (images, text, audio and video). This has coincided with advancements in microengineering and miniaturisation of electronics, leading to the instrumentation of everyday objects, commonly referred to as the ‘internet of things’^4^. In turn, various industries, including medicine, have adopted such approaches, leading to a further increase in the breadth of readily collectable data from wearable and digital technologies^5^.

This article explores the potential of common digital biomarkers and their associated characteristics of health, namely, physical function (walk), speech and acoustics (talk), cognitive (think), visual (see) and wellbeing or symptoms (feel).

## Moving from ambition to impact: why we need to embrace ‘multimodality’

At present, digital biomarker development and commercialisation is fragmented, with various research groups working on individual diseases or impairment types. There is considerable optimism, interest, and development, but there is a need to move from ambition to impact and to provide ‘value’ for patients. To achieve this, more attention must be paid to integrating and fusing multimodal data sources. Monitoring one impairment is unlikely to reveal optimal insights for the diagnosis or monitoring of complex traits or conditions (e.g., Dementia). By integrating digital biomarkers with complementary data sources, such as ‘omics’ data (multi-omics), we can usher in a new era of precision medicine guided by deep phenotyping and endotyping^2,6^.

This fusion is likely to provide richer insights on disease and has the potential to unearth unique phenotypes or signatures that would allow comparison within groups or pathological cohorts. Here we suggest the term digital biomarker ‘fingerprints’ (DBfx) to encapsulate the integration and fusion of multimodal data.

## What challenges might lie ahead?

The advent of digital measurements has opened up new avenues for comprehensively assessing health and wellness, enabling the detection of subtle changes that may aid in diagnosis and prognosis. However, to fully realise these benefits, there are a number of difficult challenges to navigate.

### Ensuring diverse and representative datasets

While digital biomarkers offer much promise, those developing these approaches need to be mindful not to widen existing health inequalities. One challenge within existing data is the significant reliance on homogenous data sets used to train and benchmark new products or methods of analysis. Indeed despite considerable validation work within consumer grade wearables, it has been highlighted only a small handful of studies that examine the impact of skin tone on accuracy rates directly^7,8^. This has raised concern that there may be significant inaccuracies within data, which may be limiting accurate health-related information for individuals with darker skin tones- exacerbating existing structural health inequalities. There is also emerging evidence that pulse oximeters may have increased error rates based on skin tone differences by devices in response to changes in activity^9^.

Overall, a renewed focus on increasing the diversity of data will yield much richer data sets and improve the health equity of digital biomarker.

### Navigating the ‘drawbacks of dimensionality’

While biomarkers provide clear opportunity and increased dimensionality of data sources, the increased array of sensors and, thus, data capture needs to be carefully considered against the broader healthcare aims, including sustainability and societal heuristics or behaviours displayed the public. AI is likely to aid in the processing and understanding of complex data, however increasingly there is broader ecosystem challenges that need to be addressed in parallel. Just because ‘we can, doesn’t mean we should’ is an essential reminder of frugality in the face of innovation. Increased ‘sensing’ and data capture have potential unintended negative impacts of data junk, storage and ultimately environmental impacts^10^.

### Authenticity, regulation and security

The pace and scale of change to generative Artificial Intelligence (AI) provides unique opportunities (integration of multimodal datasets) and challenges (verification, validation, explainability and regulation). Likewise, healthcare is increasingly delivered within community and home settings, which offers many potential social, economic and environmental benefits. The negatives of this decentralised and consumer-first approach include exposure and increased vulnerability to cyber threats or compromise of personal data. These regulatory and security challenges require closer examination from both digital biomarkers and digital health ecosystem lenses^10–12^.

## Conclusion and next steps

**Fig. 2:**
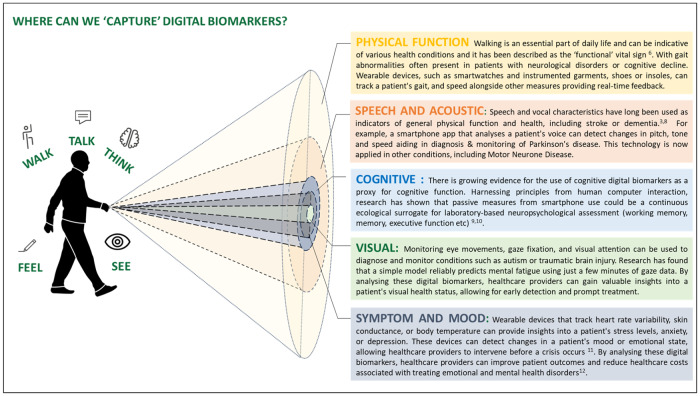
Exploring the range and scope of digital biomarkers. Parts of this figure utilises photos from following authors, all licensed under CC BY-SA https://www.stir.ac.uk/brand-bank/, https://thenounproject.com/icon/cognitive-3928976/, https://thenounproject.com/icon/time-series-2046072/, https://thenounproject.com/browse/icons/term/finger-print, Eye by Ian Anandara from https://thenounproject.com/browse/icons/term/eye, Doctor by SANB from https://thenounproject.com/browse/icons/term/doctor, Line Graph by Zach Bogart from https://thenounproject.com/browse/icons/term/line-graph.

The use of digital biomarkers in healthcare has the potential to revolutionise the diagnosis, monitoring, and treatment of various health conditions. Wearable devices, such as smartwatches, smartphones, and emerging intelligent sensors, are enabling this shift towards continuous monitoring of patients outside of the clinical setting. By integrating this data, a more complete picture of a patient’s overall health status can be gathered; the fusion of different types of traditional and digital biomarkers may provide a unique signature, termed here digital biomarker ‘fingerprint’(Fig. 2).

**Fig. 3:**
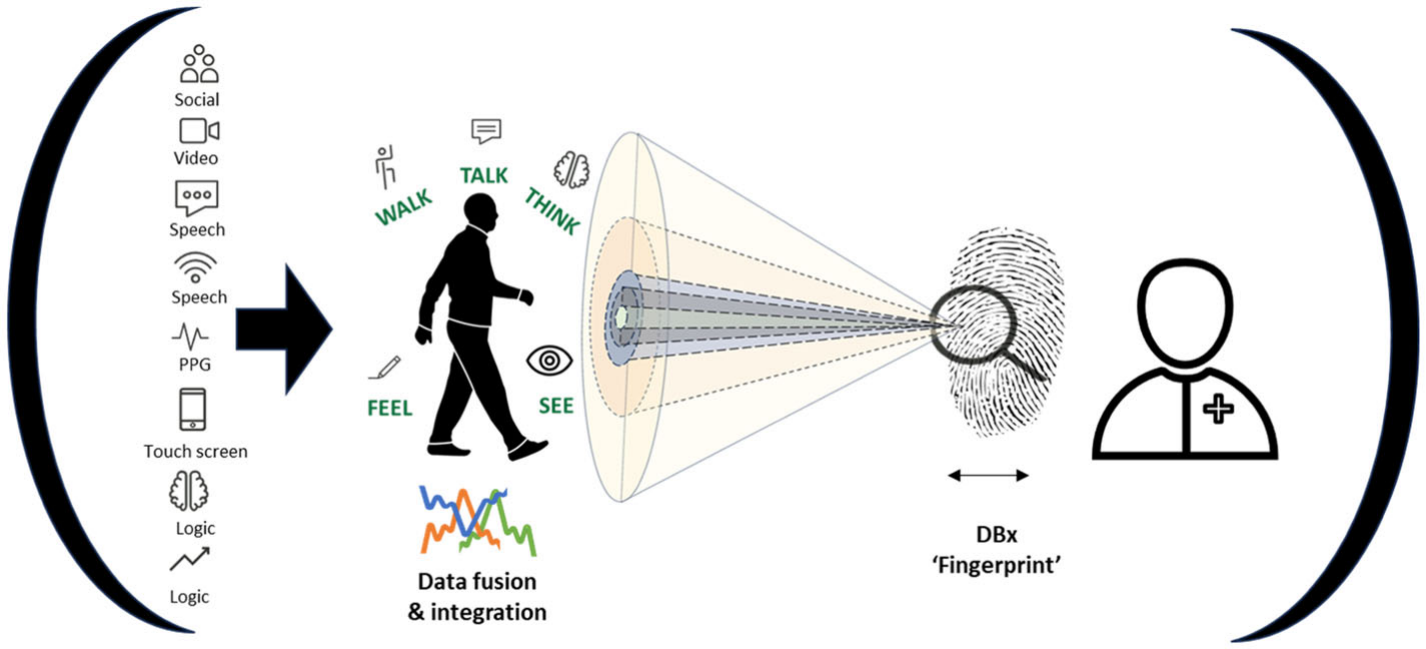
A new equation for healthcare: towards digital biomarker ‘fingerprints’ (DBfx). Parts of this figure utilises photos from following authors, all licensed under CC BY-SA. https://www.stir.ac.uk/brand-bank/, https://thenounproject.com/icon/cognitive-3928976/, https://www.stir.ac.uk/brand-bank/, https://thenounproject.com/icon/cognitive-3928976/, https://thenounproject.com/icon/time-series-2046072/, finger print by Mister Pixel https://thenounproject.com/browse/icons/term/finger-print, Eye by Ian Anandara from https://thenounproject.com/browse/icons/term/eye/.

Moving forward, to ensure value and value for patients, clinicians and health systems, the challenges of privacy, authenticity, equity and environmental sustainability must be carefully considered against the core and principal quadruple aims (care, health, cost and meaning) of healthcare^13^ (Fig. 3).
